# The Diagnosis of Gastric Disorders1Read before the Section on Medicine of the Buffalo Academy of Medicine, Jan. 10,’93.

**Published:** 1893-04

**Authors:** Allen A. Jones

**Affiliations:** Lecturer on Practice of Medicine, Instructor in Practice, and Clinical Instructor in Medicine, Medical Department, University of Buffalo; No. 436 Franklin Street


### Original Communications.

### THE DIAGNOSIS OF GASTRIC DISORDERS.[1]

By ALLEN A. JONES, M. D.,

Lecturer on Practice of Medicine, Instructor in Practice, and Clinical Instructor in Medicine, Medical Department, University of Buffalo.

Three groups of facts are considered in the diagnosis of gastric disorders:

1. The symptoms.
2. Those obtained by external examination.
3. Those obtained by direct examination.

The chief symptoms of gastric disorder, varying in number, degree and character in different cases, are pain, distress, weight, burning, eructations, regurgitation, nausea, vomiting, perversion of appetite, coated tongue, constipation, and diarrhea. More remote symptoms are anemia, vertigo, convulsions, epileptoid seizures, headache, and a host of manifestations ordinarily observed in neurasthenia.

*Pain* may be slight, or of such agonizing intensity as to cause the sufferer to cry out uncontrolledly, or to become rigid and pale. It may be diffused over the epigastrium, or localized, as in ulcer or cardialgia. It is a symptom of diagnostic import when it is a localized boring, gnawing, burning pain, and is accompanied by anemia and other evidences of gastric ulcer; or when it supervenes suddenly, with no relation to the time of eating or drinking, when it is of great degree, and is associated with extreme nervousness, as in the essential gastralgias.

In malignant disease of the stomach, pain may or may not be present. Aside from these conditions, pain is not of much diagnostic value, as it is inconstant, and is present in conditions of good gastric digestion, while absent in cases having serious gastric anomaly. When severe gastric pain exists without sufficient apparent cause, the nervous state of the patient will bear close scrutiny.

*Distress*, *weight*, and *burning* may be considered together. They are very frequent complaints of gastric disease, and exist in ever varying degrees as do the other symptoms. A vague distress predominates in one and burning in another. One or more of these may signify a loaded stomach, a stomach distended with gas,

1. Read before the Section on Medicine of the Buffalo Academy of Medicine, Jan. 10, '93.

too acid contents, or gastric hyperesthesia. They are indefinite in a diagnostic sense, and are simply expressions of a condition which remains to be determined by proper methods.

*Eructations* of air or other gases occur chiefly in certain gastric neuroses and in fermentative conditions. The most pronounced and obstinate cases are seen in neurotic individuals. Ewald suggests that in many of these cases the eructations consist of air which is taken only into the esophagus, and that the belching is not really of gastric origin. Leven, some years ago, said the eructations consisted of air ingested, and of gases from the blood and intestine, but not from fermentation of the food. The gases belched from the stomach have been analyzed several times, but no diagnostic data have been established thereby.

*Regurgitation* is suggestive of either a motor neurosis of the stomach, excessive acidity, gastrectasia, or a general neurotic state, observed in young women who present the condition called rumination or merycismus. If from a motor neurosis, it may assume the form of an undue relaxation of the cardia to normal intragastric acidity, or other stimulation; or it may occur from relaxation of the normal cardia under the influence of excessive peristalsis, or antiperistalsis, from any cause. Regurgitation of food soon after its ingestion may be so copious and persistent as to interfere with nutrition in a serious degree, but I have repeatedly found an abundance of stomach contents three or more hours after meals, which neurotic girls said they had entirely regurgitated. Occasionally, well-nourished persons inform us that they are starving to death, because they cannot retain their food, but the stomach tube reveals the truth.

*Nausea* is by no means necessarily a symptom of gastric derangement. It is, however, present in cases of malignant disease of the stomach, and in chronic gastric catarrh with a marked dyscrasia. It is more often a symptom in old people, and occurs when the tissues are poorly fed, when the cerebral and other vessels are sclerosed, and when elimination has become notably defective. As is well known, nausea and vomiting occur in the toxemia arising from renal insufficiency, and yet the mistake is not infrequently made of treating a chronic gastric catarrh with bismuth, pepsin, and dilute muriatic acid, without examining the urine. It is often a reflex as in pregnancy, pelvic disease, or eyestrain. It may be induced by grief, care, excitement, or disgusting sights. It may accompany disease of the central nervous system, or occur as a reflex from peripheral irritation. Sometimes irritating ingesta, hyperacidity, or other intragastric anomaly, will induce nausea, and when it occurs with the periodical vomiting of gastrectasia, it is an expression of gastric intolerance and poisoning from the toxines formed within the stomach. It may be caused by drugs or various toxemias. Hence, it is not of much utility in diagnosing gastric conditions, as it is present in such a diversity of disorders.

*Vomiting* may occur occasionally, persistently, or periodically. It may be projectile and easy, or difficult and painful. It is a common symptom of gastrectasia, cancer, and the gastric conditions of chronic nephritis. Vomiting may be of great diagnostic aid, or afford little information. On the whole, it may be said no symptom of gastric disease is of as great utility in diagnosis as vomiting, for the vomitus of ulcer is sometimes hemorrhagic, that of cancer often of coffee-ground character, that of dilatation with stenosis is foul, fermenting, and contains food taken days before. Then the vomitus, if sufficiently copious, may be studied microscopically and chemically, thereby shedding a flood of light upon the case. What has been said of the causation of nausea also applies to vomiting, but the latter is of much greater value as a diagnostic symptom of gastric disorders.

*Perversion of appetite* may assume the form of anorexia or bulimia in one of many degrees, or express itself in a craving for unusual articles of food. Thus many varieties of perverted appetite are encountered. Some of these are the outward expression of inward nervous, blood, or glandular disease, while others of them may be ascribed to depraved gastric innervation. In gastrectasia the appetite is often enormous and is always unsatisfied. Some cases of hyperchlorhydria desire food every two or three hours, while others with similar gastric conditions are never hungry. Perversion of the appetite affords scant diagnostic criterion.

So it is with the *tongue*. Some cases with the worst gastric conditions have the cleanest tongues, while others, with no gastric anomaly, have habitually coated tongues. A coated tongue is no certain indication of gastric disease, but may exist with such disorder. Usually, however, a coated tongue depends upon unhealthy buccal, nasal, or throat conditions, or upon toxemia of some character.

*Constipation* is present with dilatation of the stomach, with or without pyloric stenosis. Usually, however, it is more obstinate when stenosis exists. In hypotony of the gastric musculature, without dilatation, constipation is the rule.

*Diarrhea* may be one expression of impaired gastric digestion with excessive peristalsis, which has been continued to the large intestine. Lienteric diarrhea is suggestive of a gastric motor neurosis in the form of undue relaxation of the pylorus. Fermentative gastric conditions may induce gastro-intestinal catarrh, accompanied by diarrhea; and it is this form of occasional irritative diarrhea that occurs in gastrectasia.

### EXTERNAL EXAMINATION OF THE STOMACH.

Inspection, palpation, percussion, and auscultation are the methods employed in examining the stomach externally. They are practised separately and combined to determine the size, shape, position, and other conditions of the organ.

*Size.*—The most important fact to determine is the size of the stomach, as much of the therapeusis in a given case hinges upon that. Is the stomach of normal size, large, or dilated? Roughly, the lower border of the healthy stomach, in the adult, lies from one to one and a half inches above the umbilicus; a trifle higher in women than in men. It may reach the upper edge of the umbilicus, however, when distended with food, liquid, or gas, or, as is most common, a combination of these, and still be within physiological limits. When, nevertheless, the lower limit of gastric resonance is constantly at the umbilicus, whether or not the organ be filled, either megastria or moderate dilatation exists. When the lower border can be demonstrated below the umbilicus, gastrectasia is present. Sometimes the gastric contour may be seen bulging out three or four inches below the umbilicus, while palpation and percussion respectively elicit gastric splashing and resonance. If the abdominal walls are thin and relaxed, the splashing is usually easily produced by successive dips with the fingers over the gastric area from above downwards, or the patient may make it by alternate contraction and relaxation of the abdominal muscles. In the dilated stomach this is heard as far as the lower limit of the organ, and is almost characteristic to the practiced ear. If the percussion note be not satisfactory, resort to Frerich's simple means of inflating the stomach by gas evolved from the fusion of bi-carbonate of soda and tartaric acid within it, and thus the limit of resonance will be found to coincide very nearly with that of clapotage. Many methods of determining the size of the stomach are in vogue, the majority of them requiring the combined internal and external means of examination. I cannot take them up in this short paper.

*Shape and position.*—By inflating the stomach, its shape and position may be ascertained. It may be deformed, as in hour-glass contraction, vertical, horizontal, or pouched. Gastroptosis may be present, as may be demonstrated by defining the upper border of the stomach by palpation or percussion, which cannot be accomplished in health ; or by the gastrodiaphan.

*Neoplasms.*—Malignant disease of the stomach may be apparent by external examination, but often escapes the closest search for it. In a case of persistent vomiting, with evidences of gastric catarrh, and a constant absence of Hcl., five physicians beside myself, all felt a suspicious enlargement in the region of the pylorus, and we agreed that the case was one of malignant disease of the stomach. I examined the urine several times, but found no albumen nor casts. The post-mortem revealed chronic Bright's disease, but no neoplasm in or about the stomach.

In November, 1889, Dr. Max Einhorn, of New York, read before the German Medical Society of New York, an article entitled "Die Gastrodiaphanie," and he was the first to transluminate the stomach in man. Mikulicz and Trouvè illuminated the interior of the stomach about twenty years ago, but did not transluminate the gastric area in a dark room. In the New York *Medical Journal*, of December 3, 1892, Dr. Einhorn presents a communication upon Gastrodiaphany, illustrating the manner of determining the gastric areas in the normal state, in dilatation, and in gastroptosis.

### DIRECT EXAMINATION OF THE STOMACH.

If the tube cannot be used for direct examination, a small quantity of gastric contents may be withdrawn by Einhorn's stomach-bucket, which consists of a little solid silver receptacle, about the size of a thimble, to which is attached a cord. The bucket is swallowed, and after remaining a few moments in the stomach, is drawn up by means of the silk cord. By the passage of the flexible rubber stomach-tube, we learn whether strictures or diverticula of the esophagus are present or not. Strictures may be spasmodic or organic. Diverticula will usually resist the further passage of the tube, will hold only a little water, and will yield contents, notably lacking Hcl. and the products of peptonization. The stomach contents may also do this, but the tube will have passed beyond the cardia without opposition.

I will not attempt more in this paper than to give a summary of the facts learned by the study of the gastric contents. The subject is so large that a book might be written upon it.

The stomach contents are studied under the following headings: (*a*) The Food, (*b*) Mucus, (*c*) Odor, (*d*) Acidity, (*e*) Anacidity, (*f*) The Products of Gastric Digestion, (*g*) Rennet, (*h*) Absorption, and others I will not here consider.

The *food* should not remain in the stomach too long nor too short a time; it should be disintegrated and largely in a semi-solid condition in from three to four hours after its ingestion. If food is found which was eaten many hours or days before, dilatation, with or without pyloric stenosis, must exist. If, on the other hand, the stomach constantly empties itself earlier than three hours after hearty meals, an excessive gastric peristalsis, or an undue relaxation or dilatation of the pylorus exists. If the food is present unchanged, with bread simply water-soaked, and likewise meat and other substances, showing no evidence of having been acted upon by solvents, the gastric juice must be markedly deficient or wholly absent.

Much *mucus* present in the contents is evidence of gastric catarrh. It may be thick, tenacious, and ropy, or flocculent. It varies in amount in different cases and in the same case at different times. It is present as a secondary manifestation of passive congestion in chronic heart, lung, and liver diseases, in gastric cancer, gastrectasia, and many depraved conditions of the system, as chronic Bright's disease, etc.

The *odor* may be that of pronounced fermentation, as in dilatation; or of lactic acid; of acetic or butyric acids; or of odoriferous foods or drugs ingested.

The *acidity* of the gastric contents may be due to Hcl. alone, or combined with lactic and other acids; or it may entirely depend upon acid salts. The total acidity is above normal in hyperchlorhydria, or the hyperacidity may be due to lactic or other acids. If Hcl. is constantly in excess, and is present after the food has entirely disappeared from the stomach, the condition is a secretory neurosis called hyperchlorhydria, which may be present alone, or in conjunction with other neuroses. Lactic acid I find abnormally present more frequently than is generally supposed. Very frequently, indeed, in cases of gastric disorder, I find it present during the whole period of digestion. When absolutely nothing but lean beef is eaten, it may be present in large quantity four or more hours after the meal and, joined with Hcl., give rise to total acidity far above normal.

*Anacidity* of the gastric contents generally accompanies hypotony, atony, or dilatation, and there are usually present no evidences of gastric digestion. The contents may be habitually neutral in reaction, and many hours after meals the stomach empties itself into the intestine which performs the whole of digestion in a more or less perfect manner.

*Hypochlorhydria* usually exists with an over-production of lactic acid, and the presence of the latter acid seems sometimes to account for the deficient quantity of Hcl., owing to the manifest antagonism between the two acids in the stomach, but many times deficiency of Hcl. depends upon a lowered general nutrition, or is the expression of a secretory neurosis.

*Peptone and propeptone* afford fairly good evidence of gastric digestion, provided no peptonized food has been taken, and their presence is important to determine. The living stomach is the best test-oven, and when we find the end products of digestion present in fair quantity, we know peptonization has taken place. In cases of anacidity and atony, where the food is simply water-soaked and not digested, no peptone, nor propeptone, is found.

*Rennet* is absent in some cases of anacidity with apepsia, but is present in normal digestion. It is an important factor in digestion, and its presence is well worth determining.

*Absorption* is notably impaired in cases of dilatation of long standing. Sometimes several quarts of semi-fluid contents are withdrawn in these cases, and, indeed, more liquid than has been ingested during the several hours preceding. The potassium iodide test, however, is best suited in this connection, although syphonage often reveals the so-called "dyspepsia of liquids." If a few grains of potassium iodide be swallowed in a gelatine capsule, in from ten to fifteen minutes, in health, the iodine may be detected in the saliva by starch paste; whereas, in impaired absorption an hour and a half or more may elapse before the iodine appears in the saliva.

NO. 436 FRANKLIN STREET.

---

BICHLORIDE AND GERMS.—Recent researches seem to prove that when solutions of corrosive sublimate come in contact with the capsules of microörganisms, that there is a chemical reaction between the two which renders the capsule of the germ impervious, and thus protects it. It is also said that the salts of the blood or alkaline solutions acting on this substance again liberate the microörganism from the capsule formed by the bichloride.—*Courier of Medicine.*

